# In-depth analysis of the RNA editing landscape in intracranial aneurysms and its potential role in alternative splicing

**DOI:** 10.1016/j.csbj.2025.09.021

**Published:** 2025-09-15

**Authors:** Yulan Wang, Qingqing Li, Peipei Wang, Tianyi Xu, Xintong Zhao, Mingquan Ye

**Affiliations:** a School of Medical Information, Wannan Medical College, Wuhu 241002, China; b Institute of Artificial Intelligence, Hefei Comprehensive National Science Center, Hefei 230088, China; c National Genomics Data Center, China National Center for Bioinformation & Beijing Institute of Genomics, Chinese Academy of Sciences, Beijing 100101, China; d The First Affiliated Hospital of Wannan Medical College, Wuhu, China

**Keywords:** Intracranial aneurysm, RNA editing, Alternative splicing, RBP, Regulatory network

## Abstract

Intracranial aneurysm (IA) is a focal localized dilation of cerebral arteries and is a life-threatening cerebrovascular disease. Emerging evidences have emphasized the significance of post-transcriptional regulation in diseases, particularly through the two most critical regulatory layers of RNA editing (RE) and alternative splicing (AS). However, the interplay between these mechanisms and their impact on IA pathophysiology remains unclear. This study integrated multi-cohort datasets to establish a comprehensive landscape of RE in IAs. We observed a marked decrease in RNA editing levels during the transition from unruptured to ruptured aneurysms. Further analysis revealed a dual mechanism of AS by RE: direct modulation of AS via edits near splice sites that alter regulatory sequences, and indirect influence through changes in the binding affinity and specificity of RNA-binding proteins (RBPs). By constructing an RES-RBP-AS regulatory network, we identified key nodes potentially involved in IA progression via RE-mediated splicing regulation. These findings not only provide new insights into IA molecular mechanisms, but also lay a theoretical foundation for the developing therapies strategies targeting post-transcriptional regulation.

## Highlights

• Our study provided the first RNA editing landscape in intracranial aneurysms.
• Comparative analysis revealed distinct ADAR expression and RNA editing patterns between unruptured and ruptured IAs.
• RNA editing can directly regulate alternative splicing by changing the splicing regulatory sequence
• RNA editing can indirectly regulate the splicing process by altering the binding characteristics of RBPS.
• We clarified the effects of RNA editing-mediated alternative splicing regulation through the RES-RBP-AS network.

## Graphical abstract

**Figure:**
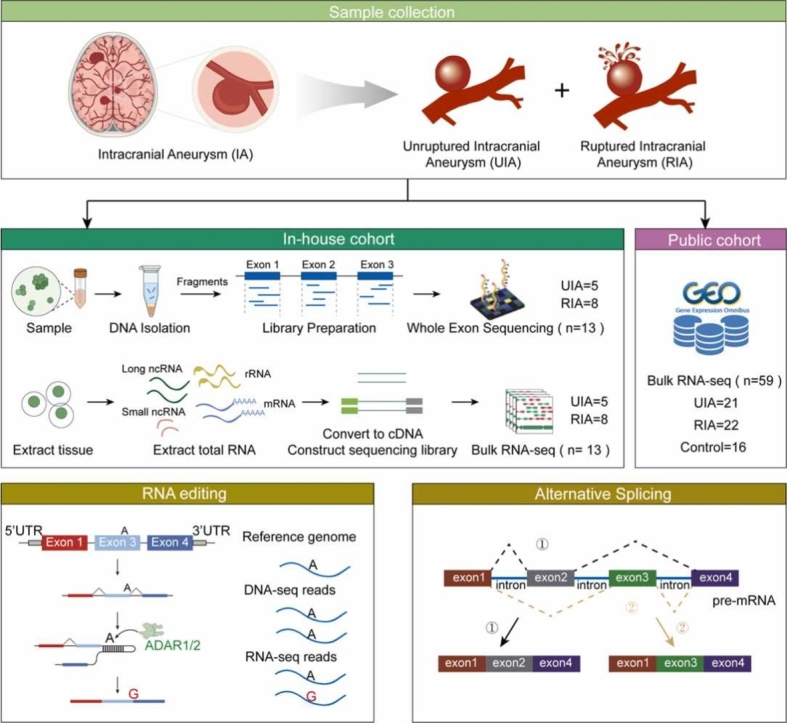

## 1 Introduction

Intracranial aneurysm (IA) is a pathological condition characterized by the abnormal protrusion of the local wall of cerebral blood vessels, with a global prevalence rate estimated at approximately 3 %[1], [2]. Once it ruptures, the fatality rate of aneurysmal subarachnoid hemorrhage (aSAH) caused by it exceeds 35 %, and half of the survivors have severe neurological dysfunction[3], [4], [5]. Despite extensive research, the molecular mechanisms driving IA formation, progression, and rupture remain incompletely elucidated. Currently, clinical rupture risk assessment mainly relies on morphological characteristics[6], [7], while the role of post-transcriptional regulation in IA pathogenesis still largely unexplored. Identifying molecular biomarkers and novel therapeutic targets related to these mechanisms is therefore urgently needed.

In recent years, post-transcriptional regulation has received increasing attention in disease pathogenesis[8], [9]. RNA editing (RE) is a post-transcriptional modification of pre-mRNA, which can dynamically change genetic information at the RNA level and significantly increase the diversity of the proteome without altering the genomic DNA sequence[10]. Alternative splicing (AS), another crucial post-transcriptional regulatory mechanism, allows a single gene to generate multiple protein isoforms with distinct functions[11], [12]. Notebly, over 95 % of the human genes undergo AS[13], [14], and its dysregulation has implicated in various diseases[15], [16], [17]. Over the past decade, the causal relationship between the dysregulation of RNA editing and tumorigenesis and development has been widely studied, and a large amount of exploratory work has also been carried out on the molecular mechanisms of IA formation and progression. However, systematic investigations into the biological role of RNA editing in IA and its potential targets remain insufficient. Significant spatiotemporal overlap between RE and AS during mRNA maturation[18], suggesting possible complex interactions between them[19], [20], [21]. Nevertheless, relationship and specific molecular mechanisms of these two regulatory processes in IA remain unresolved.

**Fig. 1:**
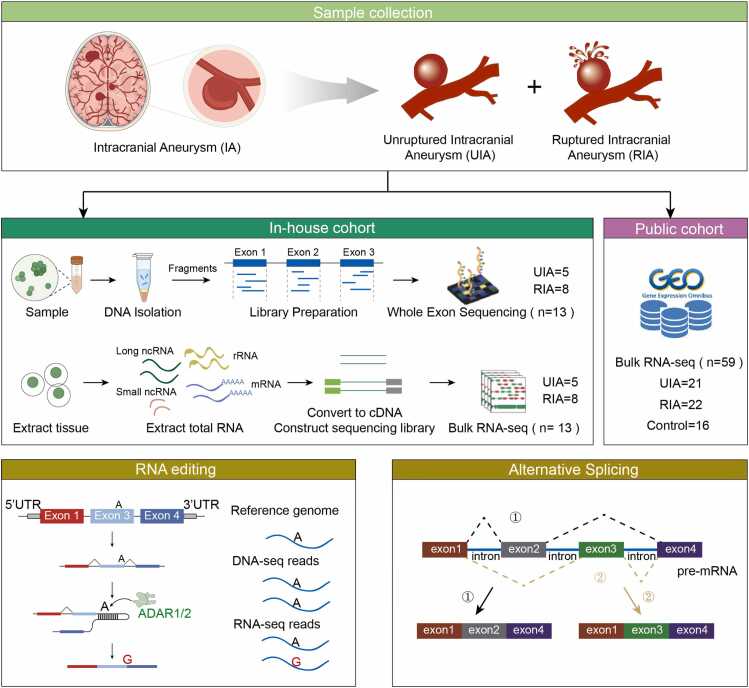
Schematic diagram of comprehensive analysis of RNA editing and alternative splicing in IA.

In this study, we integrated multi-omics data to construct the first genome-wide RNA editing atlas in IA, revealing characteristic editing patterns during the transition from unruptured intracranial aneurysm (UIA) to ruptured intracranial aneurysm (RIA). We further identified critical AS events and elucidated the intricate regulatory interplay between these two post-transcriptional modifications (Fig. 1). Our research clarifies how RE directly influences splicing by altering splice site sequences or indirectly modulates AS through RNA binding protein (RBP). Based on these findings, we successfully constructed the RES-RBP-AS regulatory network and identified key nodes potentially involved in IA through editing-mediated splicing. These research achievements not only provided a new theoretical perspective for an in-depth understanding of the molecular mechanism of aneurysm rupture, but also established an important theoretical foundation for the development of precise diagnosis and treatment strategies based on RNA regulation.

## 2 Methods

### 2.1 Sample collection

To investigate the two post-transcriptional regulatory processes of RE and AS in IA, this study integrated in-house and publicly available datasets. The in-house cohort included peripheral blood samples from 13 IA patients (8 RIA and 5 UIA) from The First Affiliated Hospital of Wannan Medical College, with matched total RNA-seq and whole-exome sequencing (WES) data. Sample collections were approved by the Human Research Ethics Committee of The First Affiliated Hospital of Wannan Medical College, and permission from the IA patients was also obtained. Additionally, publicly available RNA-seq data were downloaded from the Gene Expression Omnibus database (**GSE122897**)[22] of the National Center for Biotechnology Information (NCBI), including 22 RIA, 21 UIA, and 16 control samples. Then, we applied the combat-seq[23] to correct for batch effects across combined datasets.

The raw sequence data reported in this paper have been deposited in the Genome Sequence Archive[24] in National Genomics Data Center[25], China National Center for Bioinformation / Beijing Institute of Genomics, Chinese Academy of Sciences (GSA-Human: **HRA011925**) that are publicly accessible at https://ngdc.cncb.ac.cn/gsa-human.

### 2.2 RNA extraction

Total RNA was extracted from the tissue using TRIzol® Reagent according the manufacturer’s instructions. Then RNA quality was determined by 5300 Bioanalyser (Agilent) and quantified using the ND-2000 (NanoDrop Technologies). Only high-quality RNA sample (OD260/280 =1.8–2.2, OD260/230 ≥2.0, RQN≥6.5, 28S:18S≥1.0, >1 g) was used to construct sequencing library.

### 2.3 Library preparation and sequencing

RNA purification, reverse transcription, library construction and sequencing were performed at Shanghai Majorbio Bio-pharm Biotechnology Co., Ltd. (Shanghai, China) according to the manufacturer’s instructions (Illumina, San Diego, CA). The RNA-seq transcriptome librariy was prepared following Illumina® Stranded mRNA Prep Ligation from Illumina (San Diego, CA) using 1 μg of total RNA. Shortly, messenger RNA was isolated according to polyA selection method by oligo(dT) beads and then fragmented by fragmentation buffer firstly. Secondly doublestranded cDNA was synthesized using a SuperScript double-stranded cDNA synthesis kit (Invitrogen, CA) with random hexamer primers (Illumina). Then the synthesized cDNA was subjected to end-repair, phosphorylation and ‘A’ base addition according to Illumina’s library construction protocol. Libraries were size selected for cDNA target fragments of 300 bp on 2 % Low Range Ultra Agarose followed by PCR amplified using Phusion DNA polymerase (NEB) for 15 PCR cycles. After quantified by Qubit 4.0, paired-end RNA-seq sequencing library was sequenced with the NovaSeq X plus sequencer (2 × 150 bp read length).

### 2.4 Genomic DNA library preparation

Genomic DNA was randomly broken into fragments of 180–280 bp in length (Covaris, Massachusetts, USA). The remaining short DNA fragments were converted into blunt ends by exonuclease/polymerase activities. When a poly(A) tail was added to the short DNA fragments, they were linked by adapter oligonucleotides. The ligated DNA fragments were enriched by PCR. The library with a specific index was subjected to liquid phase hybridization with biotin-labeled probes. Then, exons were captured by magnetic beads with streptomycin, which was linearly enriched by PCR. Finally, the DNA was purified (Beckman Coulter, Beverly, USA) and quantified (Agilent Bioanalyzer 2100 system), and then, the DNA was prepared for sequencing on the Illumina NovaSeq platform.

### 2.5 High-confidence RNA editing sites (RESs) identification

The preprocessed paired-end reads were aligned to the human reference genome (hg19, Supplementary Table S1) using HISAT2[26], followed by processing with samtools to generate sorted BAM files. The identification of RNA editing sites (RESs) based on high-throughput sequencing data requires strict control of false positive interference caused by genomic variations. This study adopted a multi-step analysis process: firstly, RNA-Seq were used to preliminarily identify potential RESs through the REDItools[27]. Meanwhile, bcftools was used to call SNP on the matched WES data for its efficiency and accuracy. To ensure the accuracy of editing site detection, strict screening is carried out through the following steps: (1) remove any genomic variation sites detected in WES data; (2) exclude all known SNP included in the dbSNP database; (3) at least 3 edited reads; (4) minimum 10 read coverage[28]. Finally, ANNOVAR[29] was used to annotate the highly reliable RESs that were ultimately identified.

Differential RNA editing sites (DRESs) between UIA and RIA were identified using REDIT [30] with a β-binomial regression model. A site was considered differentially edited if the difference in average editing level between groups was ≥ 0.05 and the REDIT-LLR p-value was < 0.05.

### 2.6 Detection and analysis of alternative splicing events

rMATS[31] were used to systematically analyze RNA sequencing data to identify AS events, including skipped exons (SE), alternative 5 'splicing sites (A5SS), alternative 3' splicing sites (A3SS), mutually exclusive exons (MXE), and retained introns (RI). rMATS detects differential alternative splicing events (DASE) from RNA-seq using a modified version of the generalized linear mixture model. To ensure the reliability of the analysis results, we have set strict screening criteria: only significant difference splicing events with absolute delta percent spliced in (|ΔPSI| > 0.1) and false discovery rate (FDR < 0.05) less than 0.05 are retained for subsequent analysis[32].

### 2.7 The influence of RNA editing on alternative splicing

To assess the influence of A-to-I RNA editing events on AS, we analyzed editing events within 5 '-ss and 3′-ss regions flanking detected exons. Based on canonical splicing motifs, the 5′-ss region is a 9-mer region of 3 nt in the exon and 6 nt in the intron, whereas the 3′-ss region is a 23-mer region of 3 nt in the exon and 20 nt in the intron[33]. Changes in splice site strength due to editing were quantified using MaxEntScan[34]. We further validated splicing changes by comparing PSI values between edited and unedited samples and evaluating correlation between PSI values and editing levels to establish robust associations between A-to-I editing and splicing changes.

### 2.8 RBP binding alterations mediated by RNA editing

To identify RBP binding sites altered by RNA editing, we used RBPmap[35] to predict binding affinity in both unedited and edited sequences. Unedited sequences were extracted as ±50nt regions around the editing sites from the reference genome, while edited sequences were generated by introducing the corresponding A-to-G change. Binding sites with a change in prediction score (∆score) exceeding 20 % were considered significantly altered.

### 2.9 Gene ontology and pathway enrichment analysis

Gene ontology (GO) and Kyoto Encyclopedia of Genes and Genomes (KEGG) pathway enrichment analysis were conducted by the metascape (http://metascape.org/)[36]. GO terms, including biological process (BP), cellular component (CC), molecular function (MF), and KEGG pathways with P < 0.05 and FDR < 0.05 were considered as statistically significant.

## 3 Results

### 3.1 Characterization of high-confidence editing sites in IA

**Fig. 2:**
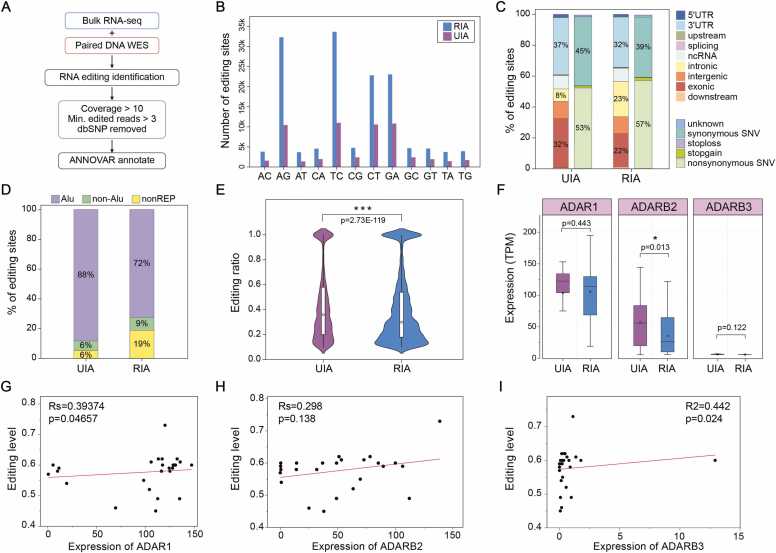
**Genome-wide characterization of RNA editing landscape in IA.** (A) Workflow of high-confidence RNA editing sites detection using integrated RNA-seq and WES data. (B) Distribution of RNA editing sites by editing type in UIA vs. RIA samples. (C) The distributions of RNA editing sites in different types of RNA regions. (D) Proportion mapped to the Alu and other elements. (E) Global RNA editing levels in UIA and RIA groups. (F) The expression level of three RNA editing enzymes: ADAR1, ADAR2 and ADAR3 across sample groups. (G-I) Correlation analysis between tADAR1 (p = 0.046), (H) ADAR2 (p = 0.138), and (I) ADAR3 (p = 0.024) with global editing levels.

Post-transcriptional RNA modification plays a key role in the pathophysiology of IA. However, the molecular mechanisms underlying RNA editing in IA rupture remains poorly understood. In this study, we integrated total RNA-Seq and WES data to systematically profile the RNA editome in UIA and RIA. Using a stringent bioinformatic pipeline, we identified high-confidence RESs (Fig. 2A). Our analysis revealed widespread RNA editing activity in IA tissues, with a significantly higher number of editing events in RIA than in UIA (Fig. 2B), suggesting a potential role for RNA editing in the rupture process. Functional annotation showed that most RESs were located in 3′UTR and exon regions, with fewer occurring in intronic and intergenic regions. Notably, approximately 55 % of editing sites resulted in non-synonymous substitutions, implying potential functional consequences on protein products (Fig. 2C). Consistent with previous studies, the majority of editing events occurred within Alu repetitive elements, with the remainder distributed in non-Alu and non-repetitive genomic regions (Fig. 2D). These findings not only affirm the robustness of our RES detection, but also provide a foundation for further mechanistic investigation into RNA editing in IA rupture.

### 3.2 ADAR regulates RNA editing events in IA

A-to-I RNA editing is the most prevalent post-transcriptional modification in mammals, is primarily catalyzed by ADAR family enzymes. To investigate the potential involvement of ADAR-mediated editing dysregulation in IA, we analyzed the expression of ADAR genes (ADAR/ADAR1, ADARB1/ADAR2, and ADARB2/ADAR3) and its correlation with global editing levels after removing batch effects from integrated RNA-seq datasets (Supplementary Fig. S1, S2). We first compared overall editing levels between ruptured (RIA) and unruptured (UIA) samples and observed a significant reduction in RIA (Fig. 2E, *paired t-test, p < 0.0001*), suggesting a potential role of RNA editing in maintaining IA stability. Subsequent analysis of ADAR expression revealed significantly lower ADAR2 levels in RIA compared to UIA (*paired t-test, p = 0.013*), with ADAR1 (*p = 0.443*) and ADAR3 (*p = 0.122*) also showing non-significant decreasing trend (Fig. 2F). Spearman correlation analysis further showed positive associations between all three ADAR enzymes and global editing activity: both ADAR1 (*p = 0.046*) and ADAR3 (*p = 0.024*) reached statistical significance, while ADAR2 exhibited a non-significant positive trend (*p = 0.138*; Fig. 2G-I). These results indicate a complex regulatory relationship between ADAR expression and RNA editing activity in IA.

### 3.3 IA in different states have different editing patterns

**Fig. 3:**
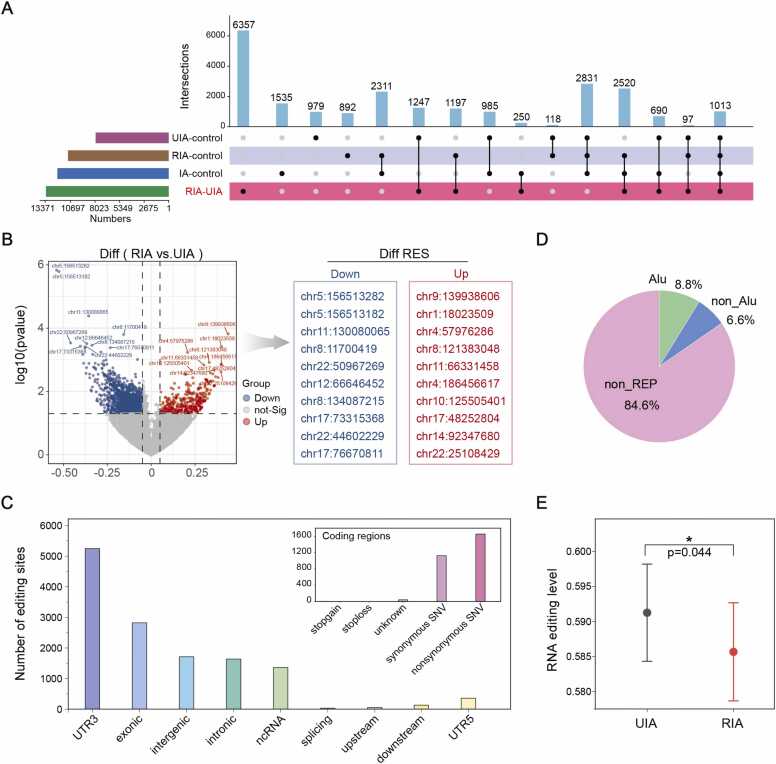
**Differential RNA editing analysis in IA.** (A) UpSet plot of RNA editing site distribution and overlap across comparison groups. (B) Volcano plot of differential RNA editing sites between RIA and UIA (FDR < 0.05, |Δ editing level| > 0.1). The right panel is the differential RNA editing events marked in the left panel. (C) Genomic distribution of differential editing sites, with inset highlighting non-synonymous versus synonymous changes in coding regions. (D) Proportion of differential editing sites mapped to the Alu and other elements. (E) Differential RNA editing levels between UIA and RIA samples.

To genome-wide identify DRESs associated with IAs, we systematically compared overall editing levels between UIA and RIA. Using generalized linear mixed model (GLMM) based on binomial distribution, we identified statistically significant DRESs. Futhermore, we also incorporated public control samples to establish four distinct comparison groups: RIA vs. control, UIA vs. control, IA vs. control, and UIA vs. RIA. Comparative analysis revealed group-specific DRES counts, with the UIA-RIA comparison showing the most pronounced differences (13,371 DRESs; Fig. 3A), predominantly exhibiting reduced editing levels (under-editing) in RIA (Fig. 3B). Functional annotation indicated that DRESs were enriched in 3′UTRs and exonic regions, with coding edits frequently introducing non-synonymous substitutions (Fig. 3C). Notably, DRESs were preferentially located in non-repetitive (non_REP) regions compared to Alu elements (Fig. 3D), and displayed significantly lower overall editing levels in RIA vs. UIA (*paired t-test, p = 0.044*; Fig. 3E). These results implicate RNA editing dysregulation as a key factor in IA rupture and stability.

### 3.4 Alternative splicing events in aneurysms

**Fig. 4:**
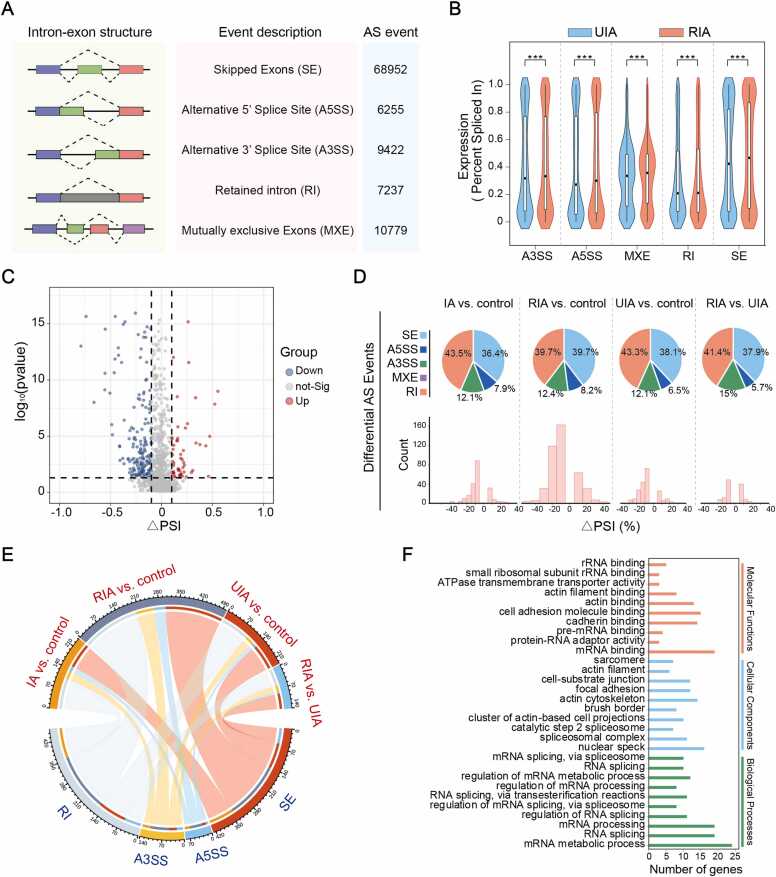
**Global profiling and functional impact of differential alternative splicing events in IA.** (A) Schematic and counts of five alternative splicing types identified by rMATS. (B) Distribution of PSI values across splicing category. (C) Volcano plot of differential splicing events between UIA and RIA (D) Proportion and counts of differential splicing events across comparison groups. (E) String graphs of the corresponding differential splicing events in different groups. (F) Gene Ontology enrichment analysis of genes containing significant differential splicing events.

To investigate the potential role of RE in IA progression through AS regulation, we comprehensively analyzed mRNA splicing profiles across integrated in-house and public datasets. Our analysis identified 102,388 AS events that differentiated UIA and RIA aneurysms (Fig. 4A). SE accounted for the majority of events (67.34 %), while A5SS were least frequent (6.11 %). Quantitative assessment using PSI metrics revealed significantly higher splicing ratios in RIA compared to UIA samples (Fig. 4B). Categorization based on ΔPSI revealed a predominance of downregulated splicing events during IA progression (Fig. 4C).

Applying stringent statistical thresholds, we identified 140 differentially expressed AS (DEAS) events (Fig. 4C). RI (n = 58,41.4 %) and SE (n = 53,37.9 %) were the most common types in RIA versus UIA comparisons, with MXE, A5SS and A3SS being less prevalent - a pattern consistent across all comparison groups (Fig. 4D-E). Functional enrichment analysis indicated that genes with DEAS events were significantly associated with RNA processing pathways, including RNA splicing, regulation of RNA splicing, and regulation of mRNA splicing through spliceosomes (Fig. 4F). These findings implicate splicing dysregulation as a key molecular mechanism in IA pathogenesis.

### 3.5 Editing sites located in the splicing site regions directly affect alternative splicing

**Fig. 5:**
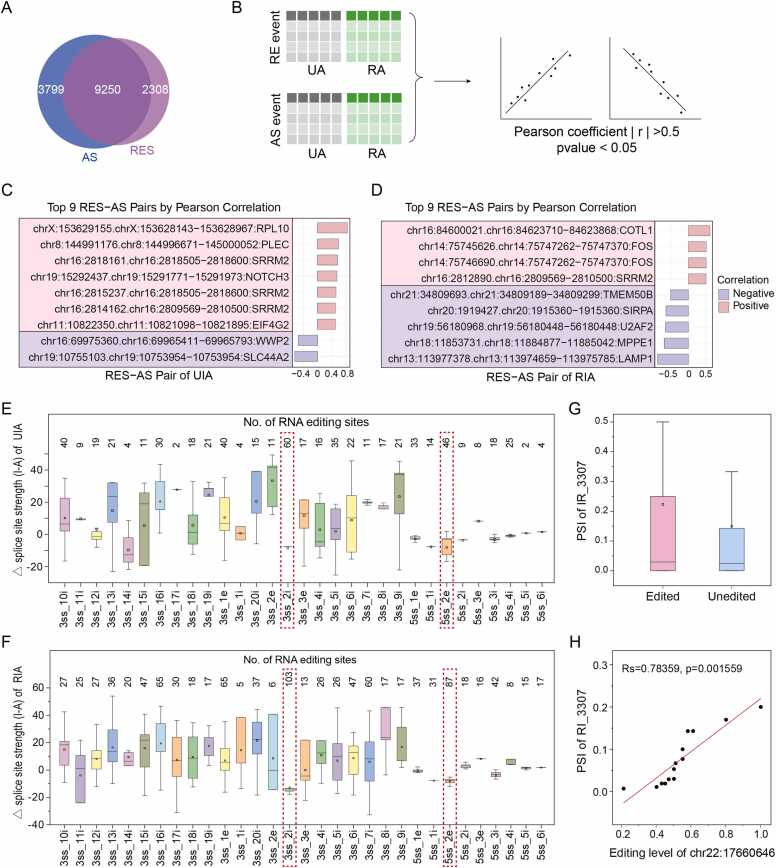
**RNA editing directly modulates alternative splicing patterns in IA.** (A) Overlap between genes with RNA editing and alternative splicing events. (B) Computational workflow for identifying correlated RES-AS pairs through integrated bioinformatics analysis. (C-D) Top correlated RES-AS pairs (C) UIA and (D) RIA, ranked by statistical significance (p < 0.05). (E-F) Impact of RNA editing on splice site strength calculated by MaxEntScan in (E) UIA and (F) RIA, comparing edited versus unedited sequences at 5'ss and 3′ss. (5’ss) 5’splice site; (3’ss) 3’ splice site; (i) intron; (e) exon. The numbers before "i" or "e" indicate the distance from the exon-intron boundary (in nucleotides). (G) Schematic representation of intron retention event (IR_3307) in ADA2 gene, preferentially occurring in edited transcripts. (H) Significant positive correlation (r = 0.78, p = 0.001) between editing frequency at chr22:17660646 and PSI of ADA2 IR_3307.

Studies have shown that RE can modulate pre-mRNA splicing by altering splice site sequences [37]. To deeply explore the direct regulatory effect of RE on AS, we systematically analyzed the influence of RE on the splicing site sequence. Among genes exhibiting AS, 9250 were also found to undergo RNA editing (Fig. 5A). For each editing site in these genes, the nearest AS event was assigned, and the RES-AS pairs with |r|> 0.5 and p < 0.05 were considered significantly associated (Fig. 5B). The top correlated RES-AS pairs in UIA and RIA revealed predominantly positive correlations in UIA and negative correlations in RIA (Fig. 5C-D).

Given that RESs near exon junctions can influence splice site strength and splicing outcomes[33], we analyzed the distribution of RESs around splice sites. Approximately 73 % of splice-proximal RESs were located near 3’ss, while 27 % were near 5’ss (Fig. 5E-F). Depending on their position, these RESs differentially affected splice site strength. For example, RES at the 3’ss-2i and 5’ss-2e positions altered core splice site sequences, potentially disrupting spliceosome recognition and reducing splice site strength. A representative example is the editing site chr:17660646, which reduced the strength of the 5’ donor splicing site (4.65–9.51 =-4.86) and was associated with increased intron retention in ADA2 (RI_3307) (Fig. 5G-H). Notably, ADA2 is associated with immune deficiency, autoinflammation, hematological abnormalities and vascular diseases. Its dysregulation is particularly prominent in neurological contexts such as transient ischemic attack and ischemic stroke[38], [39]. These findings suggest that this editing site may contribute to IA rupture by regulating ADA2 splicing.

### 3.6 RNA editing affects RBP binding to indirectly regulate alternative splicing

**Fig. 6:**
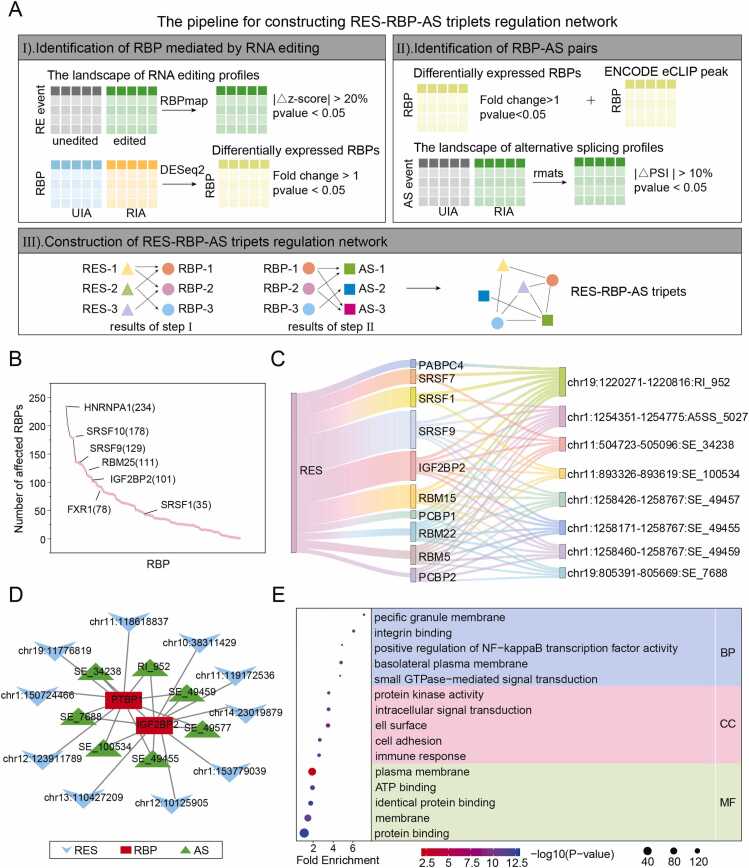
**RNA editing indirectly regulates alternative splicing through its mediated RES-RBP-AS regulatory network for IA.** (A) Computational pipeline for identifying RES-RBP-AS regulatory triplets in IA. (B) RBP binding alterations induced by RNA editing (Y-axis: number of affected RBPs; X-axis: RBP categories). (C) Sankey diagram of representative RES-RBP-AS interactions. (D) Global network visualization of significant RES-RBP-AS triplets. (E) Gene Ontology enrichment of network-associated genes.

RBP plays a key role in the regulation of splicing, with growing evidence underscoring their importance in cancers and other diseases[40]. Since RBPS typically recognize specific RNA motifs, RNA editing within these motifs may alter RBP binding affinity. We therefore hypothesized that RE events could indirectly regulate AS by affecting the binding ability of RBP. To verify this hypothesis and identify the RBP regulatory network affected by RE in IA, we implemented an integrated analytical pipeline (Fig. 6A).

This approach systematically compared RBP binding between edited and unedited sequences, identifying 125 RBPs whose binding was significantly altered by RE events (Fig. 6B). Notably, splicing regulators SRSF1 and SRSF9 - previously shown to bind AGGF1 and regulate endothelial cell function, angiogenesis and vascular development by mediating the skipping of exon 3 of the SRSF6 precursor mRNA[41]. Furthermore, RBPs such as hnRNPA1 and FXR1 have been confirmed to interact with human vascular smooth muscle cells[42], [43], [44]. FXR1 in particular regulates cytoskeletal dynamics in smooth muscle, suggesting a potential role in IA pathogenesis, which is characterized by vascular smooth muscle phenotypic modulation and cell loss.

We further constructed a genome-wide profile of AS perturbations by comparing PSI values between UIA and RIA aneurysms. By integrating eCLIP-seq data from ENCODE and correlating RBP binding changes with AS events, we identified RE-mediated RBP–AS regulatory relationships. This resulted in a network of 1948 RES-RBP-AS triples (Fig. 6C), highlighting the complexity of RE-dependent splicing regulation. Hub nodes with the highest degrees included RBP (IGF2BP2, PTBP1) and AS (SE34238, SE_49459, etc.) (Fig. 6D). Among them, IGF2BP2 has been proposed as a prognostic biomarker in IA[45], and PTBP1 promoter methylation may serve as an epigenetic predictor of IA occurrence [46]. Functional enrichment analysis o revealed that genes in the regulatory network are involved in biological processes such as cell adhesion, immune response, and intracellular signal transduction (Fig. 6E), providing mechanistic insights into how RE-driven RBP dysregulation may contribute to IA.

## 4 Discussion

IA is a disease driven by chronic inflammation, involving complex processes including vascular smooth muscle cells phenotypic transition, mural cell apoptosis, and sustained molecular inflammatory responses. Accumulating evidence underscores the critical role of post-transcriptional regulation in IA development and rupture. RNA editing, a finely tuned post-transcriptional mechanism, exhibits tissue and developmental specificity. It reflects the molecular status of vascular wall cells under pathophysiological conditions and may contribute to the transition from stable unruptured aneurysms to ruptured lesions.

In this study, we integrated RNA-seq and WES data to systematically characterize the landscape of RNA editing and alternative splicing during the progression from UIA to RIA. Using stringent bioinformatic criteria, we accurately identified RESs in both states. The number of RNA editing events was significantly higher in RIA than in UIA, suggesting that RNA editing is actively involved in aneurysm rupture. The overall editing level was significantly reduced in RIA, indicating a global dysregulation of RNA editing. Moreover, differential editing analysis identified 6357 significantly dysregulated editing sites (DRESs), whose editing levels were consistently reduced in RIA, supporting a trend toward under-editing in ruptured aneurysms.

Mechanistically, we revealed that RE can regulate AS events through both direct and indirect mechanisms. RES located near splicing junctions directly influenced splicing efficiency by changing the splice regulatory sequence. And RE can indirectly regulate AS events by altering the binding characteristics of RBPs. Through construction of a comprehensive RES-RBP-AS regulatory network, we uncovered a novel mechanism whereby RNA editing indirectly regulates splicing by altering RBP binding affinity. These findings suggest that RE and AS as core components of the post-transcriptional regulatory network, suggesting their synergistic involvement in aneurysm pathogenesis and providing potential molecular targets for therapeutic intervention.

However, several limitations should be considered. First, the relatively modest sample size may limit the statistical power to detect low-frequency editing events and reduce generalizability of findings. Future studies with larger cohorts are needed to validate and extend these observations. Second, although ADAR expression showed some association with editing levels, the relatively weak correlation suggests that additional regulatory mechanisms, such as post-translational modifications of ADAR proteins, involvement of other RNA editors, or regulation by non-coding RNAs, may contribute to the RNA editing landscape in IA. This complexity underscores the need for further investigation into the multifaceted regulation of RNA editing. Third, the regulatory relationships inferred in this study, including the RES-RBP-AS network, are primarily predictive and correlative. While our computational approaches provide strong statistical evidence for these associations, they do not establish causal relationships. The absence of experimental validation of key RES-RBP-AS interactions remains a notable limitation. Future functional studies using in vitro and in vivo models are essential to confirm the mechanistic roles of these predicted relationships.

## 5 Conclusions

In conclusion, this study provides comprehensive characterization of RNA editing events during aneurysm progression and elucidates their regulatory mechanisms on alternative splicing. These findings not only deepen our understanding of the molecular mechanism underlying IA, but also provide important clues for the development of novel diagnostic biomarkers and therapeutic targets. Subsequent studies should focus on functional validation of key editing sites and RBPs, and explore their translational potential for precise diagnosis and treatment of intracranial aneurysms.

## Funding

This work was supported by the 10.13039/501100001809National Natural Science Foundation of China (62402343), the University Synergy Innovation Program of Anhui Province, China (NO. GXXT-2022–044), the Academic Support Project for Top-notch Talents in Disciplines (Majors) of Universities in Anhui Province, China (NO. gxbjZD2022042), the Excellent Scientific Research Innovation Team Project of Universities in Anhui Province, China (NO. 2022AH010075), and the Humanities and Social Sciences Research Planning Foundation of Ministry of Education, China (NO. 22YJAZH134), Key University Science Research Project of Anhui Province (2023AH051744).

## CRediT authorship contribution statement

**Yulan Wang:** Writing – review & editing, Writing – original draft, Methodology, Investigation, Formal analysis, Data curation, Conceptualization. **Xintong Zhao:** Writing – review & editing, Validation, Investigation. **Tianyi Xu:** Writing – review & editing, Investigation. **Peipei Wang:** Writing – review & editing, Validation, Investigation. **Qingqing Li:** Writing – review & editing, Validation, Methodology, Investigation, Formal analysis, Data curation. **Mingquan Ye:** Writing – review & editing, Supervision, Project administration, Conceptualization.

## Declaration of Competing Interests

The authors declare that they have no known competing financial interests or personal relationships that could have appeared to influence the work reported in this paper.

## Data Availability

All data that support the findings of this study are available from the corresponding authors upon reasonable request.

## Footnotes

Appendix A Supplementary data associated with this article can be found in the online version at doi:10.1016/j.csbj.2025.09.021.
