# Supplementary material for: In-depth analysis of the RNA editing landscape in intracranial aneurysms and its potential role in alternative splicing
Source: Comput Struct Biotechnol J. 2025 Sep 15;27:4163–72. doi: 10.1016/j.csbj.2025.09.021 (PMC12506588; doi:10.1016/j.csbj.2025.09.021)
Supplement: Supplementary file 1 — Supplementary material [file mmc1.docx]

**In-depth analysis of the RNA editing landscape in intracranial aneurysms and its potential role in alternative splicing**

Yulan Wang^1,2,†^, Qingqing Li^1,†^, Peipei Wang^1^, Tianyi Xu^3^, Xintong Zhao^4,*^, Mingquan Ye^1,2,*^

^1^ School of Medical Information, Wannan Medical College, Wuhu 241002, China.

^2^ Institute of Artificial Intelligence, Hefei Comprehensive National Science Center, Hefei 230088, China.

^3^ National Genomics Data Center, China National Center for Bioinformation & Beijing Institute of Genomics, Chinese Academy of Sciences，Beijing 100101, China.

^4^ The First Affiliated Hospital of Wannan Medical College, Wuhu, China.

† These authors contributed equally to this work.

*To whom correspondence should be addressed: (1) Xintong Zhao, The First Affiliated Hospital of Wannan Medical College, Wuhu, China. Email: [zhaoxintong30@163.com](mailto:zhaoxintong30@163.com).

(2) Mingquan Ye, School of Medical Information, Wannan Medical College, Wuhu 241002, China; Email: [ymq@wnmc.edu.cn](mailto:yemingquan@wnmc.edu.cn).

**Supplementary Figures**

**Figure S1.** Boxplot plot before (A) and after (B) batch effect correction.

**Figure S2.** PCA plot before (A) and after (B) batch effect correction.

**Supplementary Tables**

**Table S1.** Summary of sample information for the in-house and public cohorts used in this study.

**Supplementary Figures**

**Figure S1.** Boxplot plot before (A) and after (B) batch effect correction.


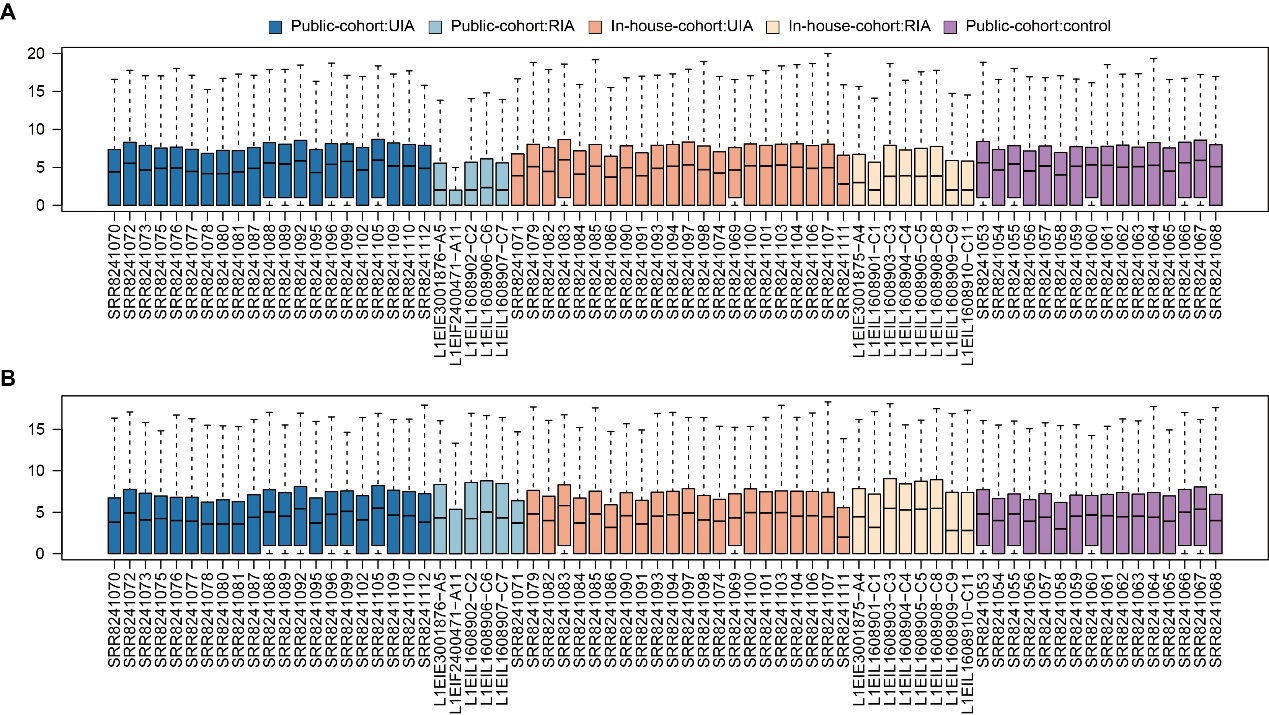


**Figure S2.** PCA plot before (A) and after (B) batch effect correction.


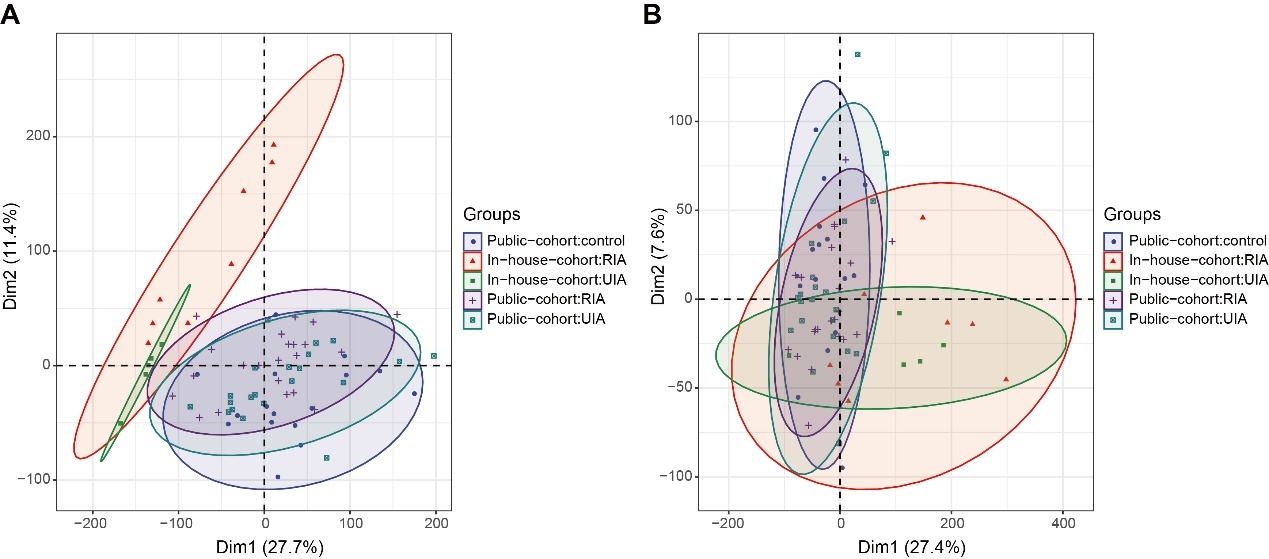


**Supplementary Tables**

**Table S1.** Summary of sample information for the in-house and public cohorts used in this study.

| **Source** | **Sample name** | **Technology** | **Sex** | **Type** | **Avg depth** | **Coverage** | **QC-passed reads** | **Alignment rate** |
| --- | --- | --- | --- | --- | --- | --- | --- | --- |
| **In-house cohort** | L1EIE3001875-A4 | RNA-seq | male | ruptured intracranial aneurysm | 2.39438 | 17.8178% | 75990937 | 93.23% |
|  | L1EIE3001876-A5 | RNA-seq | male | unruptured intracranial aneurysm | 1.76285 | 14.2166% | 87209898 | 92.09% |
|  | L1EIF2400471-A11 | RNA-seq | female | unruptured intracranial aneurysm | 2.44296 | 9.9662% | 83773441 | 91.43% |
|  | L1EIL1608901-C1 | RNA-seq | male | ruptured intracranial aneurysm | 3.0406 | 15.8293% | 94372817 | 95.16% |
|  | L1EIL1608902-C2 | RNA-seq | male | unruptured intracranial aneurysm | 2.65955 | 10.9009% | 96625922 | 95.50% |
|  | L1EIL1608903-C3 | RNA-seq | male | ruptured intracranial aneurysm | 4.70651 | 14.9367% | 89823731 | 94.87% |
|  | L1EIL1608904-C4 | RNA-seq | male | ruptured intracranial aneurysm | 2.8653 | 22.8125% | 88116034 | 94.97% |
|  | L1EIL1608905-C5 | RNA-seq | female | ruptured intracranial aneurysm | 4.07876 | 23.2372% | 81694168 | 95.54% |
|  | L1EIL1608906-C6 | RNA-seq | male | unruptured intracranial aneurysm | 2.58048 | 13.6027% | 98526020 | 95.99% |
|  | L1EIL1608907-C7 | RNA-seq | male | unruptured intracranial aneurysm | 2.51791 | 12.0730% | 110206853 | 96.35% |
|  | L1EIL1608908-C8 | RNA-seq | male | ruptured intracranial aneurysm | 4.23793 | 20.6730% | 61450655 | 95.67% |
|  | L1EIL1608909-C9 | RNA-seq | male | ruptured intracranial aneurysm | 3.93506 | 13.8842% | 91358549 | 95.04% |
|  | L1EIL1608910-C11 | RNA-seq | female | ruptured intracranial aneurysm | 3.50564 | 10.9295% | 100732803 | 95.34% |
|  | L1EIE3001870-B4 | WES | male | ruptured intracranial aneurysm | 24.5119 | 99.9683% | 40672928 | 99.79% |
|  | L1EIE3001871-B5 | WES | male | unruptured intracranial aneurysm | 26.2507 | 99.9689% | 41175652 | 99.53% |
|  | L1EIF2400479-B11 | WES | female | unruptured intracranial aneurysm | 28.0091 | 99.9734% | 38700570 | 99.90% |
|  | L1EIL1608891-D1 | WES | male | ruptured intracranial aneurysm | 31.5021 | 99.9712% | 48366465 | 99.93% |
|  | L1EIL1608892-D2 | WES | male | unruptured intracranial aneurysm | 32.4374 | 99.9713% | 48150665 | 99.94% |
|  | L1EIL1608893-D3 | WES | male | ruptured intracranial aneurysm | 32.1719 | 99.9702% | 50220321 | 99.94% |
|  | L1EIL1608894-D4 | WES | male | ruptured intracranial aneurysm | 27.6484 | 99.9720% | 38957937 | 99.92% |
|  | L1EIL1608895-D5 | WES | female | ruptured intracranial aneurysm | 29.4534 | 99.9716% | 42201862 | 99.95% |
|  | L1EIL1608896-D6 | WES | male | unruptured intracranial aneurysm | 28.9434 | 99.9717% | 42987444 | 99.94% |
|  | L1EIL1608897-D7 | WES | male | unruptured intracranial aneurysm | 30.4318 | 99.9712% | 45953968 | 99.93% |
|  | L1EIL1608898-D8 | WES | male | ruptured intracranial aneurysm | 28.8245 | 99.9725% | 40895690 | 99.93% |
|  | L1EIL1608899-D9 | WES | male | ruptured intracranial aneurysm | 32.3869 | 99.9721% | 46733450 | 99.95% |
|  | L1EIL1608900-D11 | WES | female | ruptured intracranial aneurysm | 30.746 | 99.9712% | 44973696 | 99.92% |
| **Public cohort** | SRR8241069 | RNA-seq | female | ruptured intracranial aneurysm | 0.797203 | 17.2359% | 11393583 | 90.10% |
|  | SRR8241070 | RNA-seq | male | unruptured intracranial aneurysm | 0.726019 | 13.8828% | 8902094 | 91.45% |
|  | SRR8241071 | RNA-seq | female | ruptured intracranial aneurysm | 0.566991 | 14.2694% | 5907550 | 89.71% |
|  | SRR8241072 | RNA-seq | female | unruptured intracranial aneurysm | 1.06017 | 16.3140% | 17154444 | 92.40% |
|  | SRR8241073 | RNA-seq | female | unruptured intracranial aneurysm | 0.97013 | 15.0092% | 13786730 | 89.15% |
|  | SRR8241074 | RNA-seq | male | ruptured intracranial aneurysm | 0.724173 | 13.8062% | 7727571 | 90.83% |
|  | SRR8241075 | RNA-seq | male | unruptured intracranial aneurysm | 0.76111 | 13.7597% | 9793468 | 90.01% |
|  | SRR8241076 | RNA-seq | male | unruptured intracranial aneurysm | 1.0645 | 15.5492% | 14609375 | 92.34% |
|  | SRR8241077 | RNA-seq | female | unruptured intracranial aneurysm | 0.715429 | 13.1440% | 9204365 | 85.50% |
|  | SRR8241078 | RNA-seq | female | unruptured intracranial aneurysm | 0.602595 | 13.6624% | 6280950 | 88.57% |
|  | SRR8241079 | RNA-seq | female | ruptured intracranial aneurysm | 0.952363 | 15.0574% | 13814453 | 91.74% |
|  | SRR8241080 | RNA-seq | male | unruptured intracranial aneurysm | 0.880655 | 15.4866% | 9432928 | 91.45% |
|  | SRR8241081 | RNA-seq | male | unruptured intracranial aneurysm | 0.902811 | 13.9633% | 9712058 | 88.08% |
|  | SRR8241082 | RNA-seq | male | ruptured intracranial aneurysm | 0.996731 | 14.3020% | 12430849 | 93.46% |
|  | SRR8241083 | RNA-seq | female | ruptured intracranial aneurysm | 1.17167 | 16.8221% | 20981703 | 91.93% |
|  | SRR8241084 | RNA-seq | female | ruptured intracranial aneurysm | 0.949105 | 17.9158% | 11257803 | 79.42% |
|  | SRR8241085 | RNA-seq | female | ruptured intracranial aneurysm | 0.967958 | 14.8378% | 14078635 | 93.28% |
|  | SRR8241086 | RNA-seq | male | ruptured intracranial aneurysm | 0.718434 | 13.1538% | 5555473 | 81.52% |
|  | SRR8241087 | RNA-seq | female | unruptured intracranial aneurysm | 0.769107 | 14.5969% | 10075827 | 91.66% |
|  | SRR8241088 | RNA-seq | female | unruptured intracranial aneurysm | 1.06776 | 15.9877% | 18410066 | 91.52% |
|  | SRR8241089 | RNA-seq | male | unruptured intracranial aneurysm | 0.893311 | 14.0930% | 14434143 | 89.70% |
|  | SRR8241090 | RNA-seq | female | ruptured intracranial aneurysm | 1.00084 | 14.6500% | 12353061 | 91.57% |
|  | SRR8241091 | RNA-seq | female | ruptured intracranial aneurysm | 0.793083 | 13.9646% | 8641937 | 87.28% |
|  | SRR8241092 | RNA-seq | female | unruptured intracranial aneurysm | 1.15888 | 16.6126% | 20567235 | 91.57% |
|  | SRR8241093 | RNA-seq | female | ruptured intracranial aneurysm | 0.913144 | 14.0845% | 12522816 | 94.46% |
|  | SRR8241094 | RNA-seq | female | ruptured intracranial aneurysm | 1.01017 | 14.5529% | 14177780 | 94.16% |
|  | SRR8241095 | RNA-seq | female | unruptured intracranial aneurysm | 0.90672 | 12.9634% | 8698064 | 93.28% |
|  | SRR8241096 | RNA-seq | female | unruptured intracranial aneurysm | 1.01926 | 16.3015% | 14763966 | 93.45% |
|  | SRR8241097 | RNA-seq | female | ruptured intracranial aneurysm | 1.11135 | 14.6879% | 17357494 | 93.75% |
|  | SRR8241098 | RNA-seq | male | ruptured intracranial aneurysm | 1.28748 | 13.6434% | 16503641 | 94.01% |
|  | SRR8241099 | RNA-seq | female | unruptured intracranial aneurysm | 0.906635 | 13.8883% | 14545639 | 94.49% |
|  | SRR8241100 | RNA-seq | male | ruptured intracranial aneurysm | 1.00438 | 15.8746% | 13529117 | 93.27% |
|  | SRR8241101 | RNA-seq | female | ruptured intracranial aneurysm | 0.951619 | 13.5099% | 13294940 | 93.35% |
|  | SRR8241102 | RNA-seq | female | unruptured intracranial aneurysm | 0.962222 | 15.1635% | 10908891 | 92.57% |
|  | SRR8241103 | RNA-seq | female | ruptured intracranial aneurysm | 1.25467 | 15.1954% | 17106003 | 93.49% |
|  | SRR8241104 | RNA-seq | female | ruptured intracranial aneurysm | 1.07877 | 16.6943% | 16733209 | 92.82% |
|  | SRR8241105 | RNA-seq | female | unruptured intracranial aneurysm | 1.27137 | 17.4079% | 22230050 | 93.23% |
|  | SRR8241106 | RNA-seq | female | ruptured intracranial aneurysm | 1.28706 | 15.3963% | 14949639 | 93.52% |
|  | SRR8241107 | RNA-seq | female | ruptured intracranial aneurysm | 1.22007 | 14.4518% | 16477761 | 94.40% |
|  | SRR8241108 | RNA-seq | male | ruptured intracranial aneurysm | 0.995288 | 15.4865% | 14278964 | 94.52% |
|  | SRR8241109 | RNA-seq | male | unruptured intracranial aneurysm | 1.09074 | 15.5411% | 15862534 | 94.36% |
|  | SRR8241110 | RNA-seq | female | unruptured intracranial aneurysm | 0.925406 | 12.9954% | 12554629 | 93.39% |
|  | SRR8241111 | RNA-seq | female | ruptured intracranial aneurysm | 1.47033 | 20.2029% | 13701504 | 93.12% |
|  | SRR8241112 | RNA-seq | female | unruptured intracranial aneurysm | 1.04647 | 15.2109% | 16742252 | 91.86% |
|  | SRR8241053 | RNA-seq | female | Intracranial cortical artery | 1.0588 | 14.9291% | 18915683 | 92.91% |
|  | SRR8241054 | RNA-seq | male | Intracranial cortical artery | 0.693521 | 13.7292% | 8820966 | 89.52% |
|  | SRR8241055 | RNA-seq | male | Intracranial cortical artery | 0.797442 | 14.0112% | 12696122 | 90.83% |
|  | SRR8241056 | RNA-seq | male | Intracranial cortical artery | 0.749703 | 14.0232% | 9156704 | 87.67% |
|  | SRR8241057 | RNA-seq | male | Intracranial cortical artery | 0.867544 | 14.3061% | 12163468 | 91.24% |
|  | SRR8241058 | RNA-seq | male | Intracranial cortical artery | 0.793263 | 14.2994% | 8886379 | 89.68% |
|  | SRR8241059 | RNA-seq | male | Intracranial cortical artery | 0.794726 | 13.2838% | 11908902 | 92.34% |
|  | SRR8241060 | RNA-seq | female | Intracranial cortical artery | 0.710192 | 14.8520% | 11032871 | 92.00% |
|  | SRR8241061 | RNA-seq | female | Intracranial cortical artery | 0.696201 | 13.3656% | 10684965 | 89.13% |
|  | SRR8241062 | RNA-seq | female | Intracranial cortical artery | 0.865764 | 14.1883% | 12997286 | 92.87% |
|  | SRR8241063 | RNA-seq | female | Intracranial cortical artery | 0.822205 | 14.5996% | 11591276 | 82.37% |
|  | SRR8241064 | RNA-seq | female | Intracranial cortical artery | 1.35128 | 16.1264% | 20684983 | 91.87% |
|  | SRR8241065 | RNA-seq | female | Intracranial cortical artery | 1.14539 | 16.0678% | 11063530 | 92.94% |
|  | SRR8241066 | RNA-seq | male | Intracranial cortical artery | 1.05511 | 14.8752% | 17042307 | 93.93% |
|  | SRR8241067 | RNA-seq | male | Intracranial cortical artery | 1.10302 | 16.1206% | 19616207 | 94.46% |
|  | SRR8241068 | RNA-seq | male | Intracranial cortical artery | 1.31425 | 13.3721% | 13419987 | 93.63% |
